# Structural Characteristics of Polysaccharide GP2a in *Gardenia jasminoides* and Its Immunomodulatory Effect on Macrophages

**DOI:** 10.3390/ijms231911279

**Published:** 2022-09-24

**Authors:** Pingdong Lin, Lifei Chen, Xiaojing Huang, Fangnan Xiao, Lei Fu, Dingding Jing, Jingjing Wang, Hong Zhang, Lifang Sun, Yunkun Wu

**Affiliations:** Provincial University Key Laboratory of Cellular Stress Response and Metabolic Regulation & Fujian Key Laboratory of Developmental and Neural Biology, College of Life Sciences, Fujian Normal University, Fuzhou 350117, China

**Keywords:** polysaccharide, *Gardenia jasminoides*, structural characteristics, immunomodulation, macrophages

## Abstract

Here, we elucidated the structural characteristics of a polysaccharide isolated from *Gardenia jasminoides* Ellis (labeled as GP2a) and its immunomodulatory activity. GP2a is an acidic polysaccharide with a molecular weight of 44.8 kDa, mostly comprising galacturonic acid. Methylation analysis revealed 4-Gal*p*A (74.8%) to be the major sugar residue in GP2a. Nuclear magnetic resonance analysis indicated that its main chain comprised →4)-α-D-Gal*p*A-6-OMe-(1→4)-α-D-Gal*p*A-(1→ and →4)-α-D-Gal*p*A-6-OMe-(1→2)-α-L-Rha*p*-(1→, with galactan and arabinans linked to the C-4 position of →2)-α-L-Rha*p*-(1→ residue as branched chains. Furthermore, GP2a showed no obvious toxicity to macrophages (RAW 264.7) while enhancing cell viability in a dose- and time-dependent manner. Compared with untreated cells, nitric oxide production and secretion of cytokines, such as tumor necrosis factor-α, interferon-γ, interleukin (IL)-1β, IL-6, and granulocyte macrophage colony stimulating factor, in GP2a-treated cells significantly increased after 48 h. At 300 µg/mL GP2a concentration, there was no significant difference in the cytokine levels in GP2a- and lipopolysaccharide-treated cells (the positive control). In summary, GP2a is a pectic polysaccharide with homogalacturonan and rhamnogalacturonan-I structural regions in the main chain. Based on its immunomodulatory effects in vitro, GP2a may have potential uses in functional food and medicine.

## 1. Introduction

Gardenia (*Gardenia jasminoides* Ellis), a popular shrub from the Rubiaceae family, is currently used mostly as raw material for extracting natural pigments and pharmacodynamic components. Its desiccative ripe fruit (Chinese herbal name, Zhizi) is a dual-use resource for medicine and food. According to traditional Chinese medicine (TCM), gardenia is a TCM that is bitter in taste and cold in nature and enters the heart, lung, and triple burner meridians. It is capable of clearing away heat, reducing fire, cooling blood, and eliminating stasis [1].

Polysaccharides are biomacromolecules that comprise > 10 monosaccharides with variable structures and sugar composition. Generally, they exhibit various bioactivities, including immunomodulatory, antiviral, anti-inflammatory, anti-oxidative, and anti-tumor properties [2]. Among them, immunomodulation is usually considered the most prominent and common activity of polysaccharides. The immunomodulatory mechanisms of plant polysaccharides mainly include the regulation of innate immunity, specific immunity, and immune mediators. Specifically, polysaccharides regulate the immune function by stimulating the phagocytosis of macrophages, promoting lymphocyte proliferation and transformation and antibody production, inducing cytokine secretion, and activating the complement system [3,4,5]. Further molecular and receptor level research showed that the macrophages could be activated by plant polysaccharides via recognition of and binding to specific receptors, such as Toll-like receptor 4 (TLR4), CD14, and complement receptor 3 (CR3), and thereafter induce intracellular signaling cascades to initiate immune responses and exert immunomodulatory effects [6]. Most polysaccharides from higher plants have a potential value in functional food and medicine as immunomodulators given their unique physiological activities and low toxicity.

Polysaccharides reportedly constitute 4–11% of the gardenia fruit. However, structural characterization or functional activity analysis of gardenia polysaccharides has not been conducted so far. The polysaccharide structure can be divided into primary and advanced structures; the former must be characterized to understand the latter. Moreover, polysaccharide bioactivity is closely related to their primary structure, including molecular weight, monosaccharide composition, branched degrees, and glycosidic linkage patterns. Therefore, understanding the primary structure of gardenia polysaccharides is of great significance.

In this study, we analyzed the structural features of GP2a, a homogeneous gardenia polysaccharide, through size exclusion chromatography (SEC) connected with multi-angle laser light scattering (MALLS) and refractive index (RI) detectors, high-performance anion-exchange chromatography (HPAEC), Fourier-transform infrared (FTIR) spectroscopy, scanning electron microscopy (SEM), gas chromatography–mass spectrometry (GC–MS), and nuclear magnetic resonance (NMR) spectroscopy. Furthermore, we studied the effects of GP2a on NO synthesis and cytokine secretion in RAW 264.7 cells to elucidate its possible underlying immunomodulatory role.

## 2. Results

### 2.1. Polysaccharide Purification

**Figure 1:**
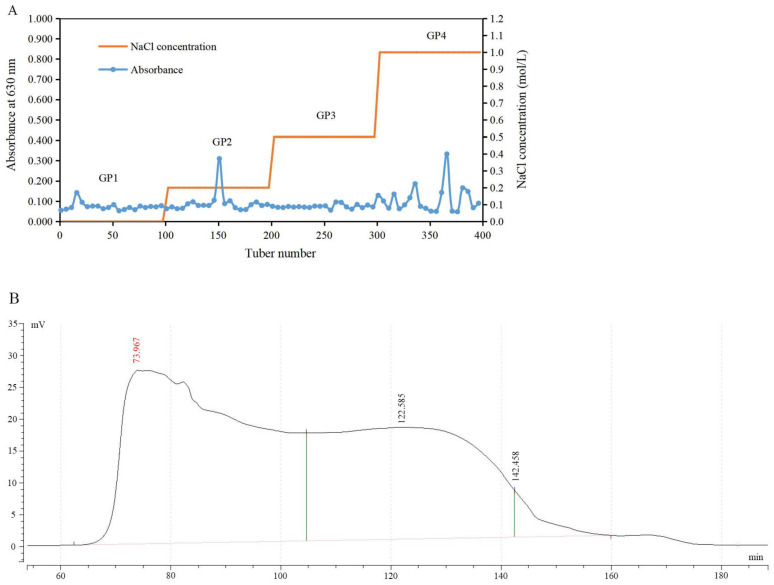
Chromatograms of polysaccharide fractionation. (**A**) Elution curve of crude polysaccharide by ion-exchange chromatography. GP1—eluted with distilled water (dH_2_O); GP2—eluted with 0.2 M sodium chloride (NaCl) solution; GP3—eluted with 0.5 M NaCl solution; GP4—eluted with 1.0 M NaCl solution. (**B**) Elution curve of GP2 by gel-filtration chromatography.

The yield of crude polysaccharide (after protein removal, decolorization and dialysis) was 3.4% *w*/*w* of defatted and dried gardenia powder. Fractionation of the crude polysaccharide by anion exchange chromatography using DEAE-Sepharose gave four fractions (Figure 1A), where GP2 had fewer stray peaks and higher total carbohydrate content compared to the other three fractions. The GP2 was collected for further purification and enrichment on Chromdex 200 PG gel-filtration column. As shown in Figure 1B, there was a single symmetrical peak between 105 and 140 min, with high total carbohydrate content. The eluted material within this time was collected, freeze-dried, and labeled as GP2a.

### 2.2. FTIR Analysis of GP2a

**Figure 2:**
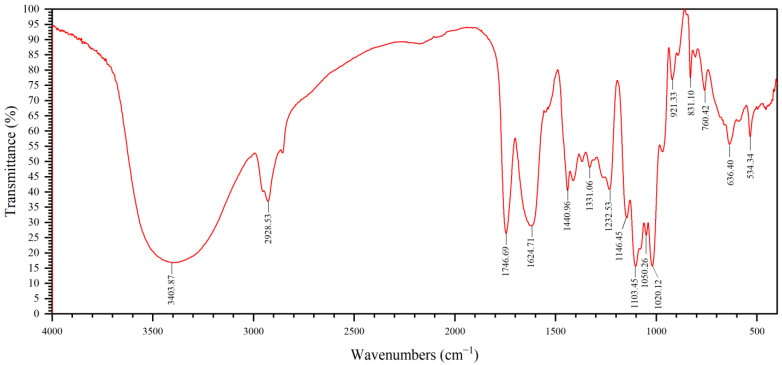
Fourier-transform infrared (FTIR) spectrum of GP2a. Bands positions are indicated in cm^−1^.

The GP2a infrared spectrum is shown in Figure 2. We detected a strong and broad band at 3403.87 cm^−1^, which is the characteristic absorption band of saccharides caused by O–H stretching vibration [7], and at 2928.53 cm^−1^, another characteristic saccharide absorption band assigned to C–H stretching vibrations. The further strong and narrow band at 1746.69 cm^−1^ corresponds to the C=O stretching vibration of esterified carboxyl groups, and that at 1624.71 cm^−1^ corresponds to the C=O asymmetric stretching vibration of the free carboxylic group [8], indicating the existence of uronic acid groups in the polysaccharide. The bands within 1146.45 cm^−1^ and 1020.12 cm^−1^ correspond to C–O–C and C–O–H stretching vibration, respectively, suggesting that the sugar rings in GP2a mainly include pyranose and furanose rings. This was also suggested by the bands at 921.33 cm^−1^ and 760.42 cm^−1^ that were caused by the asymmetric and symmetric ring vibration of D-pyranosides, respectively. Finally, the band at 831.10 cm^−1^ indicates the presence of an α-glycosidic linkage.

### 2.3. Molecular Weight and Monosaccharide Composition of GP2a

**Figure 3:**
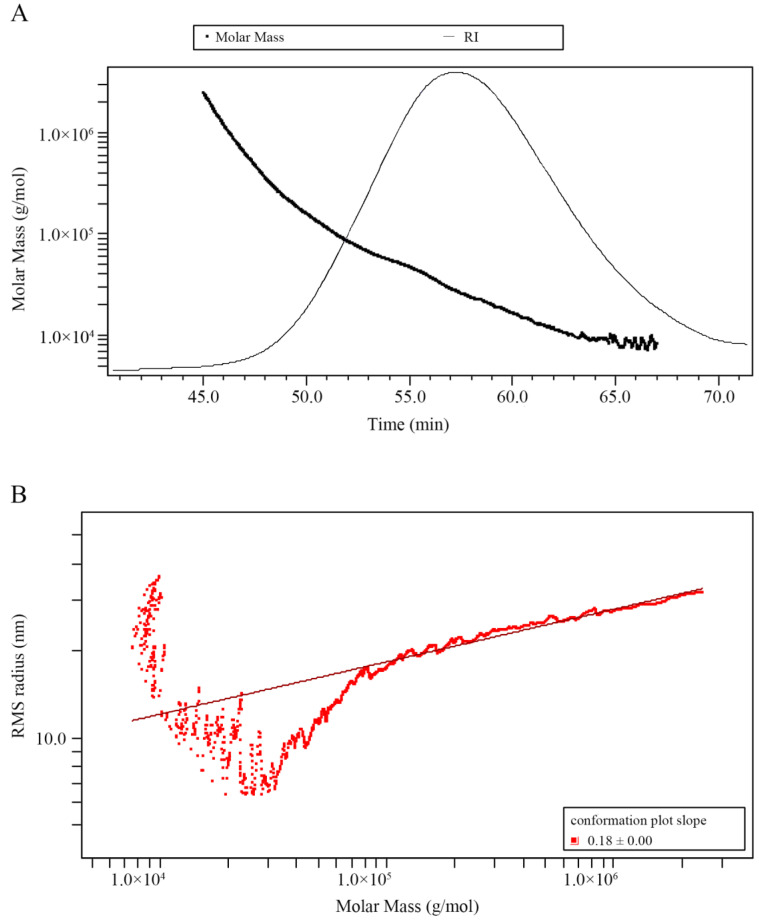
Plots of molecular weight distribution (**A**) and molecular conformation (**B**) of GP2a. (**A**) Superimposed chromatograms of GP2a obtained by size exclusion chromatography connected with multi-angle laser light scattering and refractive index (RI) detectors; the RI trace shows the signals collected by the RI detector; the molar mass trace was fitted by laser light scattering signals and RI signals, indicating molecular weight distribution. (**B**) Log–log plot of *r*_g_ versus Mw; RMS—root mean square.

A molecular weight distribution is shown in Figure 3A, the single symmetrical peak of signals recorded by RI detector indicating the homogeneity of GP2a. The weight-average molecular weight (Mw) and number-average molecular weight (Mn) of GP2a were 44.8 and 20.8 kDa, respectively. Monodisperse polymers have a polydispersity index (PDI) of 1, whereas one with wider molecular weight distribution has a PDI that is far larger than 1 [9]. The PDI of GP2a is 2.2, indicating that GP2a is a polymer with moderate dispersion. As shown in Figure 3B, the molecular conformation plot of root mean square (RMS) radius (<$r_{g}^{2}$> ^1/2^) versus molar mass (Mw) yields a line whose slope is less than 0.33 (0.18 ± 0.00), displaying that GP2a is a compact and curly spherical molecule in NaNO_3_ aqueous solution [10].

**Figure 4:**
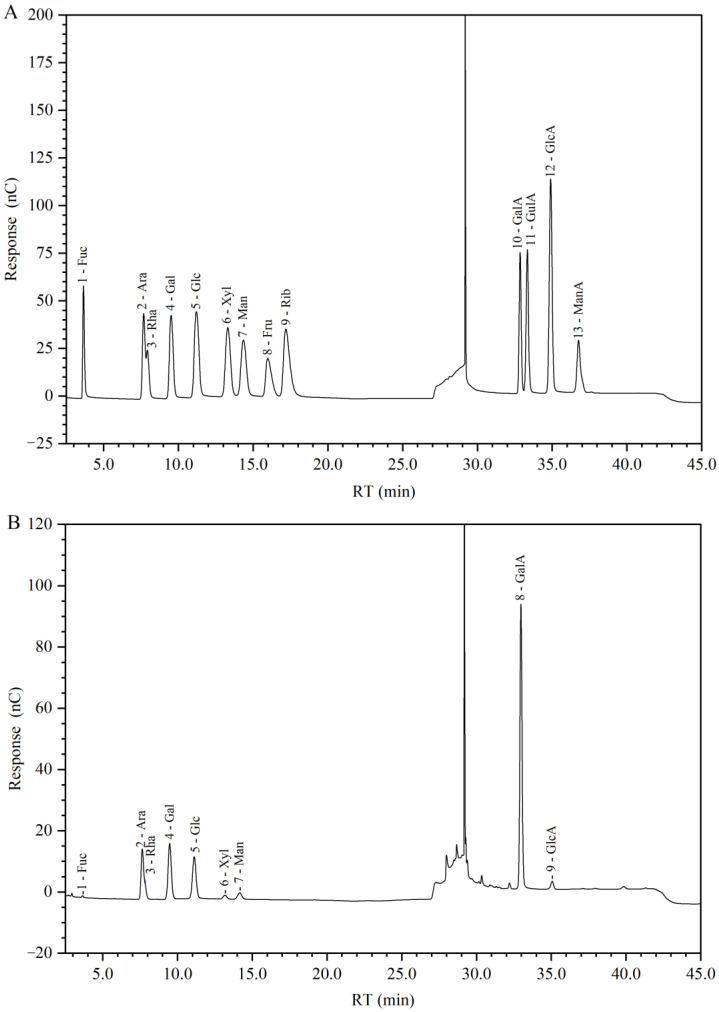
Ion chromatogram of standard monosaccharides (**A**) and GP2a hydrolysate (**B**). Peaks in (**A**): 1 is fucose (Fuc), 2 is arabinose (Ara), 3 is rhamnose (Rha), 4 is galactose (Gal), 5 is glucose (Glc), 6 is xylose (Xyl), 7 is mannose (Man), 8 is fructose (Fru), 9 is ribose (Rib), 10 is galacturonic acid (GalA), 11 is guluronic acid (GulA), 12 is glucuronic acid (GlcA), and 13 is mannuronic acid (ManA). Peaks in (**B**): 1 is Fuc, 2 is Ara, 3 is Rha, 4 is Gal, 5 is Glc, 6 is Xyl, 7 is Man, 8 is GalA, and 9 is GlcA.

In our study, the total carbohydrate content of GP2a by the phenol-sulfuric acid assay is 453 µg/mg, with glucose (Glc) as the standard. The monosaccharide composition of GP2a is shown in Figure 4 and summarized in Table 1. Galacturonic acid (GalA) was the major monosaccharide of GP2a, in a relative percentage more than 60%. Others were arabinose (Ara), galactose (Gal), Glc, rhamnose (Rha), mannose (Man), and glucuronic acid (GlcA) were found, with relative percentages in the range of 1–12%. Additionally, tiny amounts of xylose (Xyl) and fucose (Fuc) were detected. These results demonstrated that GP2a is a GalA-rich acidic polysaccharide.

### 2.4. Linkage Composition of GP2a

**Figure 5:**
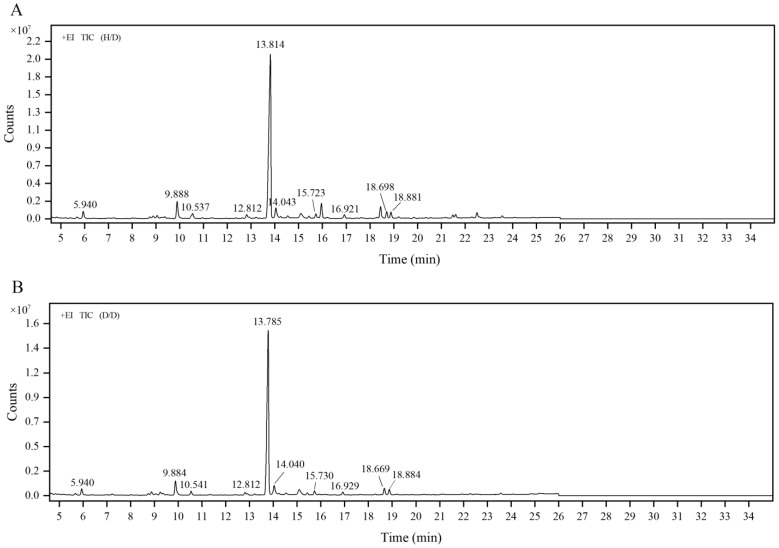
Total ion chromatograms (TIC) of GP2a derivatives. (**A**) H/D, singly deuterated sample, referring to the carboxyl groups prereduced using sodium borohydride (NaBH_4_) and secondary reduced using sodium borodeuteride (NaBD_4_) after methylation. (**B**) D/D, doubly deuterated sample, referring to the carboxyl groups reduced using NaBD_4_ before and after methylation. +EI: electron bombardment ion source.

Prior to methylation, the polysaccharide was carboxyl-reduced to its equivalent neutral sugar using carbodiimide (EDC)-sodium borohydride (NaBH_4_) and EDC-sodium borodeuteride (NaBD_4_). All partially methylated alditol acetates (PMAAs) obtained by GP2a derivatization were detected as neutral sugars by GC but distinguishable by MS, given the difference of two mass units between the carboxylic esters reduced using NaBD_4_ (6,6′-dideuterio-sugars) and the original neutral sugars. The sample PMAAs were qualitative analyzed by comparing to standard PMAAs in a local database created based on the Complex Carbohydrate Research Center (CCRC) spectral database (https://glygen.ccrc.uga.edu/ccrc/specdb/ms/pmaa/pframe.html (accessed on 12 August 2022)). By comparing the two total ion chromatograms (Figure 5), a strong peak at a retention time (RT) of 13.814 min (Figure 5A) was identified as 1,4,5-tri-O-acetyl-2,3,6-tri-O-methyl galactitol and its corresponding sugar residue was confirmed as 4-GalpA. Other sugar residues were also identified and are summarized in Table 2. The results suggest that GP2a is mainly composed of 4-GalpA. Other low percentages of GalpA (<10 mol%) were detected and they presented t-linkage, 3,4-linkage, and 4,6-linkage, demonstrating that GalA is the major residue type in GP2a, in agreement with the results from the monosaccharide composition analysis. Additionally, the terminal sugar residues also included t-Araf and t-Galp. Meanwhile, a small percentage of 5-Araf, 3-Galp, 3,4-Galp, 3,6-Galp, 4-Glcp, and 2,4-GlcpA were detected in GP2a.

### 2.5. Micromorphology Structure of GP2a

**Figure 6:**
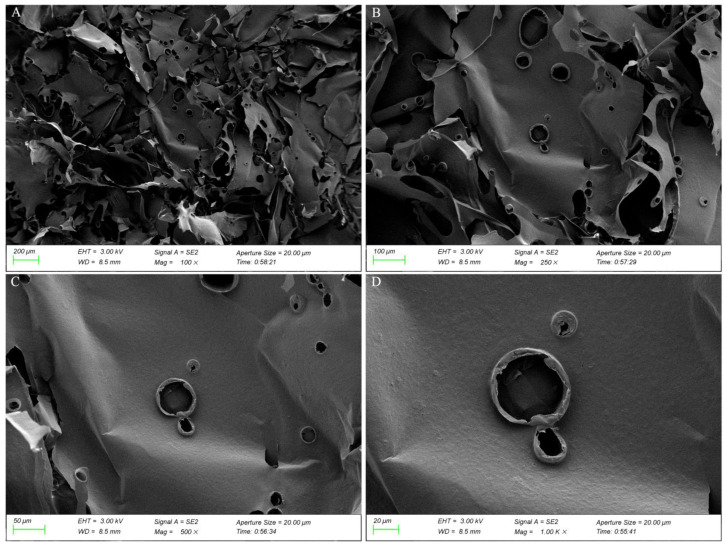
Scanning electron microscopy (SEM) images of GP2a at (**A**) 100×; (**B**) 250×; (**C**) 500×; (**D**) 1000×.

SEM images (Figure 6) at different magnifications revealed that GP2a is piled together in a lamellar or clastic form with a smooth surface as well as circular bulges and irregular apertures. This suggests the existence of a molecular repulsive force between polysaccharides with a weak intermolecular attraction, which is characteristic of most plant polysaccharides.

### 2.6. NMR Spectrum Analysis of GP2a

To further confirm the aforementioned results and obtain more detailed structural information, 1D and 2D NMR spectra were obtained.

**Figure 7:**
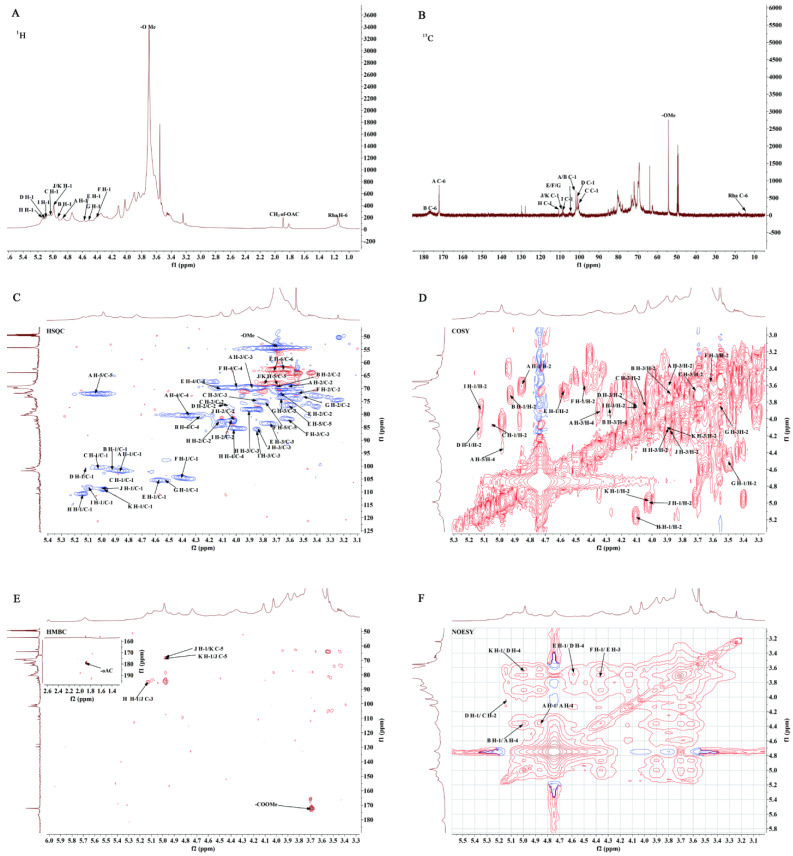
Nuclear magnetic resonance (NMR) spectra of GP2a. (**A**) ^1^H-NMR spectrum; (**B**) ^13^C-NMR spectrum; (**C**) Heteronuclear single quantum relation (HSQC) spectrum; (**D**) Correlated spectroscopy (COSY) spectrum; (**E**) Heteronuclear multiple bond correlation (HMBC) spectrum; (**F**) Nuclear Overhauser effect spectroscopy (NOESY) spectrum.

As evident from the ^1^H-NMR spectrum (Figure 7A), the proton signals were mainly concentrated in the δ 3.0–5.5 ppm region, where anomeric proton signals were found in the δ 4.5–5.5 ppm region. Other proton signals concentrated in the δ 3.0–4.5 ppm region overlapped seriously and were difficult to assign. The carbon signals showing on the ^13^C-NMR spectrum (Figure 7B) were easier to identify given fewer spectral lines compared with the ^1^H-NMR spectrum. Multiple significant anomeric carbon signals were found in the δ 90–110 ppm region that were apt for structural analysis. Based on the monosaccharide composition and methylation analysis results, the chemical shifts of the anomeric signals were determined as δ_H/C_ 4.86/101.90 ppm, δ_H/C_ 4.94/101.72 ppm, δ_H/C_ 5.03/100.78 ppm, δ_H/C_ 5.12/100.83 ppm, δ_H/C_ 4.57/105.59 ppm, δ_H/C_ 4.41/104.52 ppm, δ_H/C_ 4.50/105.84 ppm, δ_H/C_ 5.13/110.85 ppm, δ_H/C_ 5.10/108.64 ppm, δ_H/C_ 4.99/109.01 ppm, and δ_H/C_ 4.98/109.38 ppm, by analyzing the HSQC (Figure 7C) and ^1^H-^1^H COSY (Figure 7D) spectra. They were designated as A, B, C, D, E, F, G, H, I, J and K, respectively, and their chemical shifts of ^1^H and ^13^C signals are summarized in Table 3.

The major residues, such as the residue A, was analyzed as follows:

The H-1 chemical shift of residue A at 4.86 ppm was determined by the HSQC (Figure 7C) and ^1^H-^1^H COSY (Figure 7D) spectra. The other proton signals obtained from the ^1^H-^1^H COSY (Figure 7D) were δ 3.62 ppm, δ 3.89 ppm, and δ 4.35 ppm, assigned to H-2, H-3, and H-4, respectively. The C-1–C-4 signals on the sugar-ring of residue A were determined through the HMBC spectrum (Figure 7E), showing chemical shifts of 101.90 ppm, 69.44 ppm, 69.59 ppm, and 80.68 ppm, respectively. Thereby, residue A had an α-configuration.

The cross peaks of H-5 couples with C-6 were found at δ 4.98/172.30 ppm and δ 5.06/172.30 ppm in the HMBC spectrum (Figure 7E); accordingly, the C-6 chemical shift was determined at δ 172.30 ppm. Combined with the HMBC cross peak at δ 3.70/172.30 ppm, it appears that residue A had undergone esterification. As GalA is the main monosaccharide of GP2a, the signals at 176.64 ppm and δ 172.30 ppm in the ^13^C-NMR spectra (Figure 7B) were assigned to Gal*p*A. Particularly, δ_C_ 176.64 ppm is considered the typical C-6 signal of unesterified Gal*p*A, whereas δ_C_ 172.30 ppm corresponds to the typical C-6 position signal of esterified Gal*p*A. A HSQC related cross peak at δ_H/C_ 3.70/54.42 ppm (Figure 7C) shows the typical methyl ester (–COOMe) signal, further indicating the existence of methyl esterified Gal*p*A [11,12]. Moreover, GP2a appears to be a polysaccharide with high methyl esterification due to its high intensity ^1^H (δ 3.70 ppm) and ^13^C (δ 54.42 ppm) signals, as well as the high intensity C-6 position signal at δ 172.30 ppm. Multiple peaks in the region of δ_H_ 1.90–2.10 ppm suggest a possible acetylation at the O-2 and O-3 positions [13,14,15]. This was further confirmed by the HSQC related cross peak at δ_H/C_ 1.89/18.30 ppm (Figure 7C), as well as the HMBC related cross peak at δ_H/C_ 1.88/179.19 ppm (Figure 7E). However, acetyl-group signals were weak; thus, GP2a contains a low acetylation degree.

The low field shifting in C-1 and C-4 positions indicates that these two sites were substituted in residue A. Additionally, the H-5 shifted to a low field after introduction of methoxy groups. Moreover, the HSQC spectrum showed two signals of H-5 (Figure 7C) at 4.98 ppm and 5.06 ppm, suggesting that residue A is linked together with itself as well as Gal*p*A. Accordingly, residue A is inferred as →4)-α-D-Gal*p*A-6-OMe-(1→.

Coupling signals from HMBC remote correlation spectrum and NOESY spectrum served to analyze the interconnection between polysaccharide residues. As evident from the NOESY spectrum (Figure 7F), the coupling signals that H-1 of residue A is coupled with its H-4 (designated as A_H-1_/A_H-4_), confirming that residue A is linked with itself by (1→4) glycosidic bond. Based on the monosaccharide composition and linkage pattern of GP2a, we preliminarily infer that GP2a may be a pectic polysaccharide. Further, the high content of GalA and the structural features of residue A indicate that GP2a possibly contains the homogalacturonan (HG) structure. Similarly, the coupling signals designated B_H-1_/A_H-4_ and D_H-1/_C_H-2_ indicate that residue B is linked with A by a (1→4) glycosidic bond and residue D is linked with C by a (1→2) glycosidic bond, respectively. This suggests that GP2a may also contain the rhamnogalacturonan (RG)-I structure. Therefore, the GP2a backbone appears to be composed mainly of pectic HG and RG-I structural regions.

**Figure 8:**
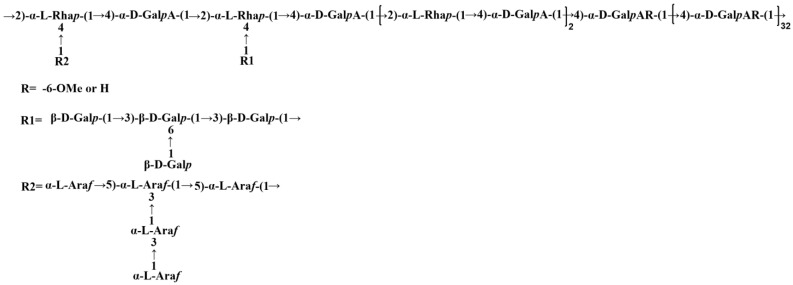
Possible repeat unit structure of GP2a.

Furthermore, other coupling signals were found in Figure 7F, where E_H-1_/D_H-4_ shows the C-4 position linkage between residues E and D, and F_H-1_/E_H-3_ shows the C-3 position linkage between residues F and E. These can be the characteristic signals of a galactan branched chain. The HMBC spectrum (Figure 7E) shows three coupling signals. where the J_H-1_/K_C-5_ and K_H-1_/J_C-5_ both indicate C-5 position linkage between residues J and K, and the H_H-1_/J_C-3_ indicates C-3 position linkage between residues H and J. The anomeric signals of residues H (δ_H/C_ 5.13/110.85 ppm), I (δ_H/C_ 5.10/108.64 ppm), J (δ_H/C_ 4.99/109.01 ppm), and K (δ_H/C_ 4.98/109.38 ppm) could be assigned to the Ara residues with differing linkage patterns, where residues H and K had been determined as α-L-Ara*f*-(1→ and →5)-α-L-Ara*f*-(1→, respectively. While the assignment of residue J has no support from the corresponding methylation data, it could be roughly inferred as →3,5)-α-L-Ara*f*-(1→ according to the typical structural features of pectic polysaccharides containing the RG-I structure and the coupling characteristics between the residues. Similarly, residue I may be →3)-α-L-Ara*f*-(1→. Therefore, GP2a possibly has another branched chain, an arabinan. Both the two branched chains are linked at the C-4 positions of Rha*p* in the RG-I structural region. The possible repeat unit structure of GP2a can be inferred as shown in Figure 8.

### 2.7. Effect of GP2a on RAW 264.7 Cell Viability

Table 4 shows the result of the Cell Counting Kit-8 (CCK-8) assay. GP2a displayed almost no toxicity against RAW 264.7 cells with increasing concentration. Within 48–72 h, the cell viability of (120–300 µg/mL) GP2a-treated cells and lipopolysaccharide (LPS)-treated cells (the positive control) were markedly enhanced compared with that of untreated cells (the blank control) (*p* < 0.01). Further, cell viability inhibition occurred with exposure to LPS for 72 h. Moreover, the viability of cells treated with GP2a (120–300 µg/mL) for 48 h were significantly higher than those obtained after 24 h (*p* < 0.01). Obviously, GP2a is efficacious for the promotion of RAW 264.7 cell viability, and accordingly the concentrations of 120, 240, and 300 µg/mL and an incubation time of 48 h were used for the following experiments.

### 2.8. Effects of GP2a on NO and Cytokine Production in RAW 264.7 Cells

**Figure 9:**
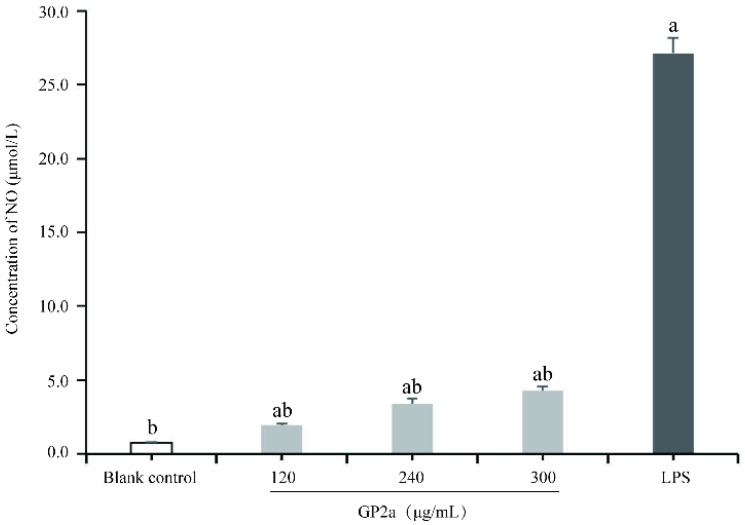
Effects of GP2a on nitric oxide (NO) production in RAW 264.7 cells. Untreated cells were used as blank control; Cells treated with different GP2a concentrations (120, 240, and 300 µg/mL) were used as samples; Cells treated with lipopolysaccharide (LPS) (2 µg/mL) were used as positive control. Letter ^a^ represents significant difference compared with the blank control (*p* < 0.05, *p* < 0.01); letter ^b^ represents significant difference compared with the positive control (*p* < 0.05, *p* < 0.01).

Figure 9 shows a significantly lower NO production in the blank control (0.754 ± 0.036 μM), than the sample groups (*p* < 0.01) and lower that in the positive control (27.135 ± 1.056 μM) (*p* < 0.01). With increasing GP2a concentration, NO production gradually increased, suggesting that the NO production of RAW 264.7 cells was GP2a concentration-dependent. The maximum NO yield (4.277 ± 0.264 μM) was obtained with 300 μg/mL GP2a treatment for 48 h. Although the high NO production with GP2a treatment was lower than that in the positive control (*p* < 0.01), it demonstrated that GP2a may have an activation effect on macrophages, which could promote NO release.

**Figure 10:**
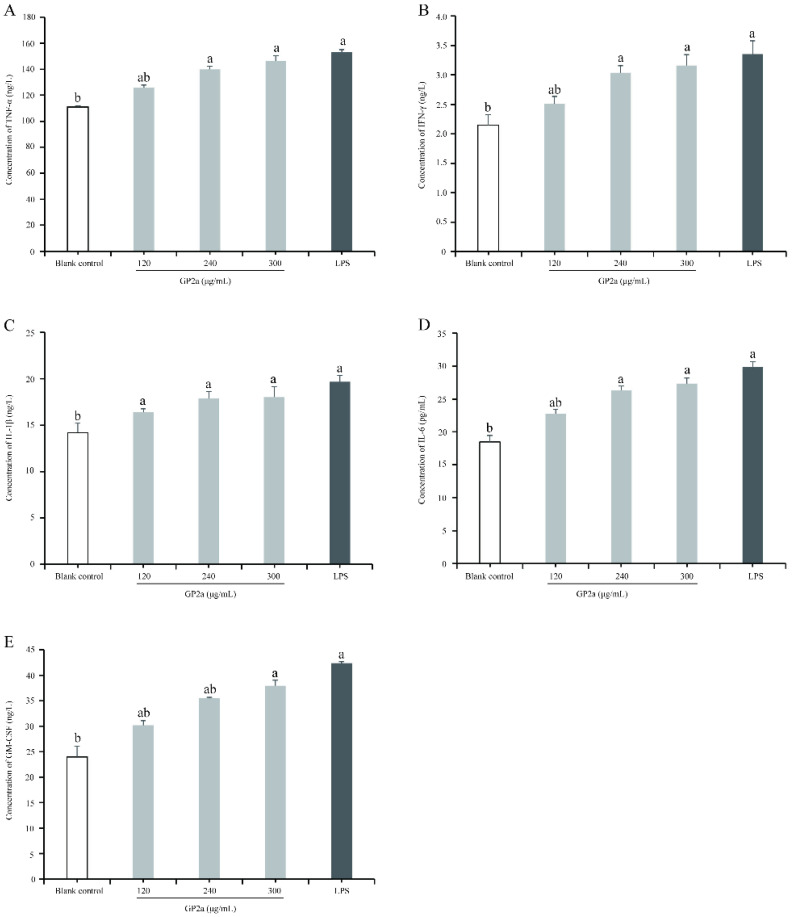
Effects of GP2a on cytokine production in RAW 264.7 cells. ELISA detection of tumor necrosis factor-α (TNF-α) (**A**), interferon (IFN)-γ (**B**), interleukin (IL)-1β (**C**), IL-6 (**D**), and granulocyte macrophage colony stimulating factor (GM-CSF) (**E**). Untreated cells were used as blank control; cells treated with different GP2a concentrations (120, 240, and 300 µg/mL) were used as sample; cells treated with lipopolysaccharide (LPS) (2 µg/mL) were used as positive control. Letter ^a^ represents significant difference compared with the blank control (*p* < 0.05, *p* < 0.01); letter ^b^ represents significant difference compared with the positive control (*p* < 0.05, *p* < 0.01).

As shown in Figure 10, the production of cytokines from the positive control and the sample groups were significantly higher than in the blank control, as indicated by markedly increased tumor necrosis factor-α (TNF-α), interferon (IFN)-γ, interleukin (IL) -1β, IL-6, and granulocyte macrophage colony stimulating factor (GM-CSF) levels (*p* < 0.05, *p* < 0.01). Particularly, the TNF-α, IFN-γ, IL -1β, IL-6, and GM-CSF levels obtained with 300 µg/mL GP2a were significantly higher than in the blank group (*p* < 0.05), but not significantly different from the positive control. GP2a seemed to induce cytokine secretion. Meanwhile, the effect of GP2a on cytokine production enhanced with increasing GP2a concentration (120–300 µg/mL).

## 3. Discussion

In this study, we unveiled the structural characteristics of GP2a and investigated its immunomodulatory activity. The results demonstrated that GP2a is an acidic pectic polysaccharide with highly methyl-esterified HG and RG-I structures, and that it shows an obvious immunomodulatory effect on macrophages in vitro. In addition, no inhibitory effect was found on macrophage viability, indicating that GP2a is safe under the current experimental conditions. Therefore, we believe that GP2a may have potential value in the application of functional food or medicine due to its immunomodulatory effects and relatively low toxicity.

Molecular weight is one of the basic parameters characterizing the properties of polysaccharides, mainly depending on its narrow or wide range [16]. The present study showed that GP2a is a homogeneous polysaccharide with a relatively low molecular weight (44.8 kDa) and has strong immunomodulatory effects on macrophages. Polysaccharides with different molecular weights also differ in their bioactivities [17]. As studies by Apostolova et al. and Wu et al. have shown, the low-molecular-weight fucoidan (<50 kDa) exhibited stronger immunomodulatory effects than the high-molecular-weight fucoidan (>100 kDa) [18,19]. In contrast, Yoo et al. and Zho et al. showed that polysaccharides with high molecular weights had better pharmacological effects than those with low molecular weights [20,21]. Nevertheless, some fractions with low molecular weights (<10 or 30 kDa) have been reported to be inactive [22]. That is to say, the molecular weight of a polysaccharide plays an important role in its bioactivities.

In this study, GP2a was found to be composed of specific HG and RG-I structural regions. The ratio of Rha and GalA, which constitute the RG-I, usually reflects the proportion of RG-I in the backbone structure. Here, the monosaccharide composition data showed that the RG-I ratio was 0.042 (Rha/GalA), demonstrating that GP2a is a pectic polysaccharide with a predominantly HG structure. This may account for the relatively low molecular weight of GP2a. Furthermore, GalA residues in the HG structure of GP2a were determined with a degree of methyl esterification (DM) of around 60%. The research by Vogt et al. found that the high-DM (74%) pectins activating Toll-like receptors were much more pronounced than the low-DM (30%) pectins, indicating that DM can influence the immunostimulatory characteristics of pectins [23]. Klosterhoff et al. showed that the pectic polysaccharide from acerola with a high DM (86%) has intracellular antioxidant activity on the murine fibroblast cell line (NIH/3T3) by reducing hydrogen peroxide (H_2_O_2_)-induced cytotoxic effects and reactive oxygen species (ROS) levels [24]. Apparently, DM influences the bioactivity of pectin polysaccharides, and accordingly, we can infer that the relatively high methyl-esterified GP2a may have similar bioactivities. In addition, branched chains in the RG-I region are possibly the main structures for complement activation, supported by the finding that complement fixation activity could be enhanced by hydrolyzed products from pectic polysaccharides containing RG-I structure (with AG-I and/or AG-II branches) [25,26]. Thus, the RG-I structure is crucial for the immunomodulatory activity of pectic polysaccharides. A complete skeleton of pectic polysaccharides is key to their immunomodulatory effects. Based on our data, the HG and RG-I structures of GP2a potentially explain its immunomodulatory activity.

The intervention conditions of polysaccharides are usually determined by examining their effects on cell viability. The data from our study indicate that RAW 264.7 cells have good viability when exposed to GP2a at concentrations between 7.5 and 300 µg/mL, but a significant difference still could be found between the sample groups and the positive control even when the GP2a concentration was 300 µg/mL. This suggests that GP2a concentrations within 300 µg/mL are non-toxic to cells. In addition, the cell viability could be effectively improved by appropriately prolonging the intervention time in a specific GP2a concentration range. In brief, GP2a exerts a positive effect on macrophages in a concentration- and time-dependent manner. LPS can stimulate host immune effector cells to produce NO and cytokines, such as TNF-α, IFN-γ and IL, thereby inducing immunological stress [27,28]. As one of the main immune effector cells, macrophages are key target cells for LPS and polysaccharides [29]. In this study, immune-stressed model was established by LPS-treated RAW 264.7 cells and used as positive control. NO release by activated RAW264.7 cells has been commonly used to evaluate the immunomodulatory activity of compounds. We found that GP2a significantly induced NO release from RAW 264.7 cells in vitro, and increased TNF-α, IFN-γ, IL-1β, IL-6, and GM-CSF levels in the meantime; this demonstrates that GP2a may activate macrophages and could produce an immune response similar to that induced by LPS. The results of the present study are similar to those from research into pectic polysaccharides with highly methyl-esterified HG or RG-I that could exert immunomodulatory effects by directly inducing macrophage activation [30,31,32,33]. However, the nonspecific induction of the immune system is only one of its main mechanisms, the specific pathway by which GP2a exerts its immunomodulatory effects remains to be studied.

The bioactivity of polysaccharides is not only dependent on their primary structure but is also closely related to their specific spatial structure [34,35]. Our study preliminarily learned about the molecular chain conformation of GP2a from the log–log plot of *r*_g_ versus Mw, which showed that GP2a has a relatively aggregated molecular chain morphology, which is closest to the spherical conformation. The aggregation state of polysaccharide molecular chain could be affected by both the length and the branching degree of side chains. Thus, GP2a’s spherical conformation is possibly related to its highly branched galactan and arabian side chains, with branching degree of 8.3 [(Ara + Gal)/Rha] [36]. However, the exact relationship between the molecular chain conformation of the polysaccharide and its bioactivity remains unclear, as well as the mechanism of action. Therefore, it is necessary to conduct an in-depth study into the advanced structure of the polysaccharide and its structure-activity relationship in order to elucidate its specific structural features which help macrophages to exert immunomodulatory activity. These studies are still in the development stage due to the complexity and polymorphism of polysaccharide spatial structures, as well as the relatively limited technology for characterizing the dynamic changes of the advanced structures of polysaccharides [37]. Future research should focus on addressing these issues.

## 4. Materials and Methods

### 4.1. GP2a Preparation

Before extraction, gardenia desiccative ripe fruits were crushed and soaked in 95% ethanol overnight for defatting. The defatted gardenia were dried until ethanol was completely removed and extracted once with distilled water at a 1:20 ratio (*w*/*v*) at 80 °C for 50 min by ultrasonic-assisted extraction. After extraction, the supernatant was filtered and concentrated by rotary evaporator to less than half of the original volume at 65 °C, followed by adding three volumes of ethanol and maintained at 4 °C for 24 h. The precipitate was collected after centrifugation and dried at 60 °C in an oven; then, it was subjected to protein removal (by repeated freezing–thawing at −20 °C and room temperature, and centrifugation), decolorization (using D941 adsorption resin), dialysis (using dialysis bag with cut-off 3500 Da), and freeze-drying to obtain the crude polysaccharides. Thereafter, the crude polysaccharides were separated by DEAE-sepharose FF ion-exchange chromatography with dH_2_O and a gradient of NaCl. The separated components were further purified and enriched using Chromdex 200 PG gel-filtration chromatography with dH_2_O. The purified polysaccharide was collected and dialyzed, concentrated, and freeze-dried.

### 4.2. GP2a Identification

#### 4.2.1. FTIR Scanning

To study the functional groups of GP2a, 2 mg of GP2a was weighed and treated with potassium bromide, i.e., KBr pellet. The treated sample was then scanned using FTIR (Nicolet iZ-10, Thermo Fisher Scientific, Waltham, MA, USA) at a wavelength of 4000–400 cm^−1^.

#### 4.2.2. Molecular Weight Determination

The GP2a sample was dissolved in 0.1 M NaNO_3_ aqueous solution containing 0.02% NaNO_3_ at a final concentration of 1 mg/mL, then filtered through a 0.45 μm microporous filtering film. The homogeneity and molecular weight of various fractions were measured using SEC-MALLS-RI. The molecular weight of various fractions in the sample solution were measured on a DAWN HELEOS-II laser photometer (He-Ne laser, λ = 663.7 nm, Wyatt Technology Co., Santa Barbara, CA, USA) equipped with three tandem columns (300 × 8 mm, Shodex OH-pak SB-805, 804 and 803; Showa Denko K.K., Tokyo, Japan), which were held at 45 ℃ with a model column heater, at a flow rate of 0.4 mL/min. The differential RI detector (Optilab T-rEX, Wyatt Technology Co., Santa Barbara, CA, USA) was simultaneously connected to obtain the concentration of fractions and the dn/dc value. The dn/dc value of the fractions in the NaNO_3_ aqueous solution was determined to be 0.141 mL/g. ASTRA 6.1 software (Wyatt Technology) was used to acquire and process data.

#### 4.2.3. Monosaccharide Composition Analysis

The GP2a samples (5.1 ± 0.1 mg) were hydrolyzed with trifluoroacetic acid (TFA) (2.0 M) at 121 °C for 2 h in a sealed tube. Next, the sample was dried using nitrogen (N_2_) and then washed with methanol. We repeated methanol washing 2–3 times. The residue was redissolved in deionized water and filtered through 0.22 μm microporous filtering film for measurement. The treated sample was analyzed and detected by HPAEC on a Dionex™ CarboPac™ PA-20 (150 × 3.0 mm, 10 μm) anion-exchange column connected with a pulsed amperometric detector (PAD; Dionex ICS 5000 system). A total of 5.0 μL of the treated sample was injected into the column (30 °C) for gradient elution using a mixed solvent system (mobile phase A—0.1 M NaOH; mobile phase B—0.1 M NaOH and 0.2 M NaAc) at a flow rate of 0.5 mL/min. Gradient program was performed as follows: A/B (95∶5, *v*/*v*) at 0 min, A/B (80∶20, *v*/*v*) at 30.1 min, A/B (60∶40, *v*/*v*) at 45 min, A/B (95∶5 *v*/*v*) at 45.1 min, A/B (95∶5, *v*/*v*) at 60 min. Data were acquired on the ICS5000 (Thermo Scientific), and processed by Chromeleon 7.2 CDS (Thermo Scientific).

Standard sugars (Fuc, Ara, Rha, Gal, Glc, Xyl, Man, Fru, Rib, GalA, GulA, GlcA, and ManA) were using for quantification by external standard method.

#### 4.2.4. Methylation Analysis

Polysaccharide was pre-treated by the following derivatization [38]: A total of 10.0 mg GP2a sample was dissolved in ultrapure water, then 1.0 mL EDC was added and reacted for 2 h. The sample was split into two equal parts for reduction with 1.0 mL NaBH_4_ and 1.0 mL NaBD_4_ (as the control), respectively. After 3 h, 0.1 mL acetic acid was used to terminate the reaction. The reduced sample and 1.0 mg NaOH were dissolved in anhydrous dimethyl sulfoxide (DMSO) and reacted for 30 min. Then, the solution was alkylated with methyl iodide for 1 h. This chemical reaction was terminated with 1.0 mL ultrapure water and extracted with 2.0 mL CH_2_Cl_2_. The methylated sample was obtained from the CH_2_Cl_2_ phase and dried with a rotary evaporation, and then hydrolyzed by TFA (2.0 M). Aqueous ammonia (2.0 M) and NaBD_4_ (1.0 M) were successively added to the hydrolyze sample for reduction. The reaction was terminated using acetic acid and then acetylated with acetic anhydride. Finally, the samples were extracted with CH_2_Cl_2_ again and then analyzed by GC-MS (Agilent 7890A-5977B, Agilent Technologies Inc., Santa Clara, CA, USA) fitted with an electron bombardment ion source and a MassHunter workstation, on a BPX70 (25 m × 0.22 mm × 0.25 μm, TRAJAN) column.

The operation conditions are as follows: 10:1 of the split ratio and 140 °C as initial temperature for 2.0 min; then, the temperature was increased to 230 °C in steps of 3 °C/min for 3 min. All detections were performed under a full-scan acquisition mode with the mass scanning range of 30–600 (*m*/*z*).

#### 4.2.5. SEM Observation

The GP2a sample was pressed and stuck on the sample stage with conductive adhesive. A field emission SEM (Merlin Compact, Carl Zeiss AG, Jena, Germany) equipped with two second electron detectors (in-lens Duo & Everhart Thornley) was used to observe the surface morphology of the GP2a sample under the STEM mode. All images of the GP2a sample were acquired and analyzed by Zeiss ZEN Connect software.

#### 4.2.6. NMR Spectroscopy

The GP2a sample was placed in an NMR tube and dissolved with 0.5 mL D_2_O (99.96%). High-resolution one-dimensional (1D) NMR (^1^H-NMR, ^13^C-NMR) and two-dimensional (2D) NMR (^1^H-^1^H COSY, HSQC, HMBC, NOESY) spectra were recorded by a Bruker AVANCE HD III 600MHz NMR spectrometer operating at room temperature. Chemical proton shifts in HDO (δ_H_ = δ 4.70 ppm) and methyl carbon shifts in TMSP (δ_C_ = −1.80 ppm) were used for ^1^H and ^13^C calibration, respectively.

### 4.3. Immunomodulatory Effect of GP2a on RAW 264.7 Cells

#### 4.3.1. Cell Culture

RAW 264.7, a murine macrophage cell line, was obtained from the American Type Culture Collection (ATCC TIB-71). Cells were cultured with Dulbecco’s Modified Eagle Medium containing 10% fetal bovine serum and 1% penicillin–streptomycin (Procell Life Science & Technology Co., Ltd., Hubei, China), and incubated at 37 °C and 5% CO_2_, with subculturing every 2–3 days [39].

#### 4.3.2. Cell Viability Assay

RAW 264.7 cells were seeded in 96-well culture plates at a density of 5 × 10^4^ cells/mL (100 µL/well) and incubated overnight. Thereafter, a culture medium with GP2a at various concentrations (7.5–300 µg/mL) was added and incubated for 24, 48, or 72 h. Meanwhile, untreated cells were used as blank control and the cells treated with 2 µg/mL LPS (MedChemExpress) were used as positive control. Following intervention, 10 µL CCK-8 solution (MedChemExpress) were added to each well followed by incubation at 37 °C for 45 min. The absorbance was detected using an ELISA reader (ReadMax-1900; Flash Spectrum Biotechnology Co., Ltd., Shanghai, China) by measuring the optical density value at 450 nm.

#### 4.3.3. Determination of NO and Cytokine Production in RAW 264.7 Cells

To detect the production of NO and cytokines, RAW 264.7 cells were seeded in 6-well culture plates at a density of 5 × 10^4^ cells/mL overnight. According to the cell viability assay, cells were treated with GP2a at 120, 240, and 300 µg/mL for 48 h, respectively. The blank control and positive control were the same as above. After incubation, supernatants were collected and mixed immediately with Griess Reagent (1:1 of Griess Reagent I and II, Beyotime Biotechnology, Co., Ltd., Shanghai, China) at room temperature. The absorbance at 540 nm was read using an ELISA reader. To calculate the NO concentration, a standard curve was plotted by determining the absorbance of a standard NaNO_2_ solution (at 0, 1, 2, 5, 10, 20, 40, 60, 100 μM) at 540 nm. Additionally, the secretion levels of TNF-α, IFN-γ, IL-1β, IL-6, and GM-CSF were all measured using commercial regents (MSK Biotechnology, Co., Ltd., Wuhan, China). These assays were performed as per manufacturer’s instructions.

### 4.4. Statistical Analysis

All data were analyzed by one-way analysis of variance using IBM SPSS 20.0 software; all values are expressed as means ± standard deviation. A value of *p* < 0.01 was considered to indicate a statistically significant difference, whereas a value of *p* < 0.05 was considered to indicate a trend.

## Figures and Tables

**Table 1:** Monosaccharide composition of GP2a.

Monosaccharide	Relative Percentage * (%)
GalA	63.1 ± 1.7
Ara	11.1 ± 0.6
Gal	10.9 ± 0.5
Glc	7.4 ± 0.3
Rha	2.7 ± 0.1
Man	2.2 ± 0.2
GlcA	1.2 ± 0.0
Xyl	0.8 ± 0.1
Fuc	0.2 ± 0.0

GalA—galacturonic acid; Ara—arabinose; Gal—galactose; Glc—glucose; Rha—rhamnose; Man—mannose; GlcA—glucuronic acid; Xyl—xylose; Fuc—fucose. Relative percentage *, calculated on total composition content basis. The values given represent the mean of three independent measurements.

**Table 2:** Methylation analysis result of GP2a.

Linkage Pattern	Methylated Sugar	Molecular Weight (Da)	Relative Molar Percentage (%)	RT (min)
t-Ara*f*	1,4-di-O-acetyl-2,3,5-tri-O-methyl arabinitol	279	3.1	5.940
5-Ara*f*	1,4,5-tri-O-acetyl-2,3-di-O-methyl arabinitol	307	3.0	10.537
t-Gal*p*	1,5-di-O-acetyl-2,3,4,6-tetra-O-methyl galactitol	323	3.4	9.888
3-Gal*p*	1,3,5-tri-O-acetyl-2,4,6-tri-O-methyl galactitol	351	1.4	12.812
3,4-Gal*p*	1,3,4,5-tetra-O-acetyl-2,6-di-O-methyl galactitol	379	0.7	15.723
3,6-Gal*p*	1,3,5,6-tetra-O-acetyl-2,4-di-O-methyl galactitol	379	1.9	18.881
t-Gal*p*A	1,5-di-O-acetyl-2,3,4,6-tetra-O-methyl galactitol (6,6′-dideuterio)	325	3.4	9.888
4-Gal*p*A	1,4,5-tri-O-acetyl-2,3,6-tri-O-methyl galactitol (6,6′-dideuterio)	353	74.8	13.814
3,4-Gal*p*A	1,3,4,5-tetra-O-acetyl-2,6-di-O-methyl galactitol (6,6′-dideuterio)	381	0.7	15.723
4,6-Gal*p*A	1,4,5,6-tetra-O-acetyl-2,3-di-O-methyl galactitol (6,6′-dideuterio)	381	2.0	18.698
4-Glc*p*	1,4,5-tri-O-acetyl-2,3,6-tri-O-methyl glucitol	351	4.3	14.043
2,4-Glc*p*A	1,2,4,5-tetra-O-acetyl-3,6-di-O-methyl glucitol (6,6′-dideuterio)	381	1.3	16.921

RT—retention time. Data obtained by analyzing the H/D and D/D total ion chromatograms and mass spectra.

**Table 3:** ^1^H NMR and ^13^C NMR chemical shifts of GP2a recorded in D_2_O.

Sugar Residue		Chemical Shift (ppm)								
			1	2	3	4	5	6a	6b	CH_3_ of OMe
A	→4)-α-D-Gal*p*A-6-OMe-(1→	H	4.86	3.62	3.89	4.35	5.05/4.98			3.70
		C	101.90	69.44	69.59	80.68	72.27	172.30		54.42
B	→4)-α-D-Gal*p*A-(1→	H	4.94	3.70	3.90	4.27	n.d.			
		C	101.74	69.59	69.80	80.68	n.d.	176.64		
C	→2)-α-L-Rha*p*-(1→	H	5.03	4.06	3.84	3.66	3.90	1.15		
		C	100.78	77.30	74.29	74.13	69.78	15.64		
D	→2,4)-α-L-Rha*p*-(1→	H	5.12	4.09	3.84	3.65	3.90	1.15		
		C	100.83	77.05	74.29	81.72	69.78	15.64		
E	→3)-β-D-Gal*p*-(1→	H	4.57	3.68	3.75	4.10	3.61	3.64	3.74	
		C	105.59	71.81	83.57	69.75	76.08	62.30		
F	→3,6)-β-D-Gal*p*-(1→	H	4.41	3.56	3.61	4.02	3.82	3.80	3.94	
		C	104.52	71.59	81.88	70.12	74.81	71.07		
G	β-D-Gal*p*-(1→	H	4.50	3.50	3.66	3.91	3.61	3.64	3.74	
		C	105.84	72.54	73.65	69.96	76.66	62.30		
H	α-L-Ara*f*-(1→	H	5.13	4.12	3.89	4.02	3.69/3.74			
		C	110.85	83.57	78.14	85.26	64.89			
K	→5)-α-L-Ara*f*-(1→	H	4.98	4.03	3.90	4.01	3.69/3.78			
		C	109.38	82.46	78.14	85.47	68.43			

NMR—nuclear magnetic resonance; D_2_O—deuterium oxide. n.d.—not detected.

**Table 4:** Cell viability of RAW 264.7 cells.

Groups		Cell Viability (%)		
		24 h	48 h	72 h
Blank control		100.0 ± 0.0 ^b^	100.00 ± 0.0 ^b^	100.0 ± 0.0 ^b^
Samples	GP2a 7.5 (µg/mL)	99.4 ± 2.4 b	103.6 ± 1.3 ^ab^*	104.8 ± 1.6 ^ab^*
	GP2a 15 (µg/mL)	101.3 ± 1.4 ^b^	103.1 ± 1.5 ^b^	103.5 ± 2.4 ^b^
	GP2a 30 (µg/mL)	105.3 ± 1.5 ^ab^	102.2 ± 2.5 ^b^	104.0 ± 1.4 ^b^
	GP2a 60 (µg/mL)	106.2 ± 1.1 ^a^	104.9 ± 2.3 ^ab^	102.1 ± 2.4 ^b^
	GP2a 120 (µg/mL)	103.3 ± 1.5 ^b^	107.9 ± 2.2 ^ab^*	105.6 ± 2.4 ^ab^
	GP2a 240 (µg/mL)	102.5 ± 1.9 ^b^	110.2 ± 1.8 ^ab^*	110.3 ± 3.55 ^ab^*
	GP2a 300 (µg/mL)	104.3 ± 1.2 ^ab^	115.1 ± 2.8 ^ab^*	108.0 ± 3.0 ^ab^
Positive control (2 µg/mL LPS)		109.8 ± 5.5 ^a^	118.6 ± 2.1 ^a^*	45.9 ± 3.2 ^a^*

Blank control—untreated cells; Samples—cells treated with different GP2a concentrations (7.5, 15, 30, 60, 120, 240, 300 µg/mL); Positive control—cells treated with lipopolysaccharide (LPS) (2 µg/mL). Intervention time—24 h, 48 h, and 72 h. Letter ^a^ represents significant difference compared with the blank control (*p* < 0.05, *p* < 0.01); letter ^b^ represents significant difference compared with the positive control (*p* < 0.05, *p* < 0.01); symbol * represents significant difference compared with 24 h (*p* < 0.01).

## References

[1] Wang S.C., Tseng T.Y., Huang C.M., Tsai T.H. (2004). Gardenia herbal active constituents: Applicable separation procedures. J. Chromatogr. B Analyt. Technol. Biomed. Life Sci..

[2] Chen Y., Yao F., Ming K., Wang D., Hu Y., Liu J. (2016). Polysaccharides from Traditional Chinese Medicines: Extraction, Purification, Modification, and Biological Activity. Molecules.

[3] Özdemir R.B.Ö., Özdemir A.T., Sarıboyacı A.E., Uysal O., Tuğlu M.İ., Kırmaz C. (2019). The investigation of immunomodulatory effects of adipose tissue mesenchymal stem cell educated macrophages on the CD4 T cells. Immunobiology.

[4] Merecz-Sadowska A., Sitarek P., Śliwiński T., Zajdel R. (2020). Anti-Inflammatory Activity of Extracts and Pure Compounds Derived from Plants via Modulation of Signaling Pathways, Especially PI3K/AKT in Macrophages. Int. J. Mol. Sci..

[5] Cambeiro-Pérez N., González-Gómez X., González-Barreiro C., Pérez-Gregorio M.R., Fernandes I., Mateus N., de Freitas V., Sánchez B., Martínez-Carballo E. (2021). Metabolomics Insights of the Immunomodulatory Activities of Phlorizin and Phloretin on Human THP-1 Macrophages. Molecules.

[6] Yin M., Zhang Y., Li H. (2019). Advances in Research on Immunoregulation of Macrophages by Plant Polysaccharides. Front. Immunol..

[7] Ji X., Yan Y., Hou C., Shi M., Liu Y. (2020). Structural characterization of a galacturonic acid-rich polysaccharide from Ziziphus Jujuba cv. Muzao. Int. J. Biol. Macromol..

[8] Manrique G.D., Lajolo F.M. (2002). FT-IR spectroscopy as a tool for measuring degree of methyl esterification in pectins isolated from ripening papaya fruit. Postharvest. Biol. Technol..

[9] Yang J.S., Mu T.H., Ma M.M. (2018). Extraction, structure, and emulsifying properties of pectin from potato pulp. Food Chem..

[10] Wyatt P.J. (1993). Light scattering and the absolute characterization of macromolecules. Anal. Chim. Acta.

[11] Petersen B.O., Meier S., Duus J.Ø., Clausen M.H. (2008). Structural characterization of homogalacturonan by NMR spectroscopy-assignment of reference compounds. Carbohydr. Res..

[12] Yang J., Wen L., Zhao Y., Jiang Y., Tian M., Liu H., Liu J., Yang B. (2018). Structure identification of an arabinogalacturonan in Citrus reticulata Blanco ‘Chachiensis’ peel. Food Hydrocolloids.

[13] Patova O.A., Smirnov V.V., Golovchenko V.V., Vityazev F.V., Shashkov A.S., Popov S.V. (2019). Structural, rheological and antioxidant properties of pectins from *Equisetum arvense* L. and *Equisetum sylvaticum* L.. Carbohydr. Polym..

[14] Perrone P., Hewage C.M., Thomson A.R., Bailey K., Sadler I.H., Fry S.C. (2002). Patterns of methyl and O-acetyl esterification in spinach pectins: New complexity. Phytochemistry.

[15] Renard C.M.G.C., Jarvis M.C. (1999). Acetylation and methylation of homogalacturonans 1, optimisation of the reaction and characterisation of the products. Carbohydr. Polym..

[16] Zeng P., Li J., Chen Y., Zhang L. (2019). The structures and biological functions of polysaccharides from traditional Chinese herbs. Prog. Mol. Biol. Transl. Sci..

[17] Han B., Baruah K., Cox E., Vanrompay D., Bossier P. (2020). Structure-Functional Activity Relationship of β-Glucans From the Perspective of Immunomodulation: A Mini-Review. Front. Immunol..

[18] Apostolova E., Lukova P., Baldzhieva A., Katsarov P., Nikolova M., Iliev I., Peychev L., Trica B., Oancea F., Delattre C. (2020). Immunomodulatory and Anti- Inflammatory Effects of Fucoidan: A Review. Polymers.

[19] Wu N., Wen Z., Xiang X., Huang Y., Gao Y., Qu Y. (2015). Immunostimulative Activity of Low Molecular Weight Chitosans in RAW264.7 Macrophages. Mar. Drugs.

[20] Yoo H.J., You D.J., Lee K.W. (2019). Characterization and Immunomodulatory Effects of High Molecular Weight Fucoidan Fraction from the Sporophyll of Undaria pinnatifida in Cyclophosphamide-Induced Immunosuppressed Mice. Mar. Drugs.

[21] Zhu Y., Yao Y., Gao Y., Hu Y., Shi Z., Ren G. (2016). Suppressive Effects of Barley β-Glucans with Different Molecular Weight on 3T3-L1 Adipocyte Differentiation. J. Food Sci..

[22] Flórez-Fernández N., Torres M.D., González-Muñoz M.J., Domínguez H. (2018). Potential of intensification techniques for the extraction and depolymerization of fucoidan. Algal. Res..

[23] Vogt L.M., Sahasrabudhe N.M., Ramasamy U., Meyer D., Pullens G., Faas M.M., Venema K., Schols H.A., Vos P. (2016). The impact of lemon pectin characteristics on TLR activation and T84 intestinal epithelial cell barrier function. J. Funct. Foods.

[24] Klosterhoff R.R., Bark J.M., Glänzel N.M., Iacomini M., Martinez G.R., Winnischofer S.M.B., Cordeiro L.M.C. (2018). Structure and intracellular antioxidant activity of pectic polysaccharide from acerola (Malpighia emarginata). Int. J. Biol. Macromol..

[25] Zou Y.F., Barsett H., Ho G.T., Inngjerdingen K.T., Diallo D., Michaelsen T.E., Paulsen B.S. (2015). Immunomodulating pectins from root bark, stem bark, and leaves of the Malian medicinal tree Terminalia macroptera, structure activity relations. Carbohydr. Res..

[26] Ho G.T., Zou Y.F., Wangensteen H., Barsett H. (2016). RG-I regions from elderflower pectins substituted on GalA are strong immunomodulators. Int. J. Biol. Macromol..

[27] Van der Meide P.H., Schellekens H. (1996). Cytokines and the immune response. Biotherapy.

[28] Cavaillon J.M. (2018). Exotoxins and endotoxins: Inducers of inflammatory cytokines. Toxicon.

[29] Chakraborty I., Sen I.K., Mondal S., Rout D., Bhanja S.K., Maity P., Maity G.N. (2019). Bioactive polysaccharides from natural sources: A review on the antitumor and immunomodulating activities. Biocatal. Agric. Biotechnol..

[30] Cao J., Tang D., Wang Y., Li X., Hong L., Sun C. (2018). Characteristics and immune enhancing activity of pectic polysaccharides from sweet cherry (Prunus avium). Food Chem..

[31] Wang H., Bi H., Gao T., Zhao B., Ni W., Liu J. (2018). A homogalacturonan from Hippophae rhamnoides L. Berries enhance immunomodulatory activity through TLR4/MyD88 pathway mediated activation of macrophages. Int. J. Biol. Macromol..

[32] Huang L., Zhao J., Wei Y., Yu G., Li F., Li Q. (2021). Structural characterization and mechanisms of macrophage immunomodulatory activity of a pectic polysaccharide from Cucurbita moschata Duch. Carbohydr. Polym..

[33] Georgiev Y.N., Paulsen B.S., Kiyohara H., Ciz M., Ognyanov M.H., Vasicek O., Rise F., Denev P.N., Yamada H., Lojek A. (2017). The common lavender (Lavandula angustifolia Mill.) pectic polysaccharides modulate phagocytic leukocytes and intestinal Peyer’s patch cells. Carbohydr. Polym..

[34] Zhang B., Leung W.K., Zou Y., Mabusela W., Johnson Q., Michaelsen T.E., Paulsen B.S. (2014). Immunomodulating polysaccharides from Lessertia frutescens leaves: Isolation, characterization and structure activity relationship. J. Ethnopharmacol..

[35] Patel B.K., Campanella O.H., Janaswamy S. (2013). Impact of urea on the three-dimensional structure, viscoelastic and thermal behavior of iota-carrageenan. Carbohydr. Polym..

[36] Wei C., Zhang Y., Zhang H., Li J., Tao W., Linhardt R.J., Chen S., Ye X. (2019). Physicochemical properties and conformations of water-soluble peach gums via different preparation methods. Food Hydrocol..

[37] Xu Y., Wu Y., Sun P., Zhang F., Linhardt R.J., Zhang A. (2019). Chemically modified polysaccharides: Synthesis, characterization, structure activity relationships of action. Int. J. Biol. Macromol..

[38] Pettolino F.A., Walsh C., Fincher G.B., Bacic A. (2012). Determining the polysaccharide composition of plant cell walls. Nat. Protoc..

[39] Lategan K.L., Walters C.R., Pool E.J. (2019). The effects of silver nanoparticles on RAW 264.7. Macrophages and human whole blood cell cultures. Front. Biosci..

